# Rapid high-yield expression and purification of fully post-translationally modified recombinant clusterin and mutants

**DOI:** 10.1038/s41598-020-70990-3

**Published:** 2020-08-28

**Authors:** Sandeep Satapathy, Rebecca A. Dabbs, Mark R. Wilson

**Affiliations:** 1 Illawarra Health and Medical Research Institute, School of Chemistry and Molecular Bioscience, University of Wollongong, Wollongong, NSW 2522 Australia; 2 Molecular Horizons Research Institute, University of Wollongong, Rm 313, Building 42 (Molecular Horizons), Northfields Avenue, Wollongong, NSW 2522 Australia; 3 Burnett Institute, 85 Commercial Road, Melbourne, VIC 3004 Australia

**Keywords:** Biochemistry, Biological techniques, Biotechnology

## Abstract

The first described and best known mammalian secreted chaperone, abundant in human blood, is clusterin. Recent independent studies are now exploring the potential use of clusterin as a therapeutic in a variety of disease contexts. In the past, the extensive post-translational processing of clusterin, coupled with its potent binding to essentially any misfolded protein, have meant that its expression as a fully functional recombinant protein has been very difficult. We report here the first rapid and high-yield system for the expression and purification of fully post-translationally modified and chaperone-active clusterin. Only 5–6 days is required from initial transfection to harvest of the protein-free culture medium containing the recombinant product. Purification to near-homogeneity can then be accomplished in a single affinity purification step and the yield for wild type human clusterin is of the order of 30–40 mg per litre of culture. We have also shown that this system can be used to quickly express and purify custom-designed clusterin mutants. These advances dramatically increase the feasibility of detailed structure–function analysis of the clusterin molecule and will facilitate identification of those specific regions responsible for the interactions of clusterin with receptors and other molecules.

## Introduction

Clusterin (CLU) is the first reported abundant extracellular mammalian chaperone and promiscuously interacts with misfolded proteins to stabilise them in a soluble form^1–3^. This activity is similar to that of the small heat shock proteins and places CLU in the class of chaperones known as “holdases”^3^. CLU is the best known of a currently small family of extracellular chaperones that have been proposed to neutralise and clear extracellular body fluids of toxic aggregating proteins, and thus play a key role in protecting the body from serious diseases that arise from dysfunctions in proteostasis^4^. For example, it was has been shown that CLU reacts with a variety of Aβ oligomers, ranging from monomers to 50-mers, to inhibit their aggregation and toxicity^5^, and that changes in the CLU gene pose one of the highest known risk factors for Alzheimer’s disease^6,7^. Under conditions of endoplasmic reticulum (ER) stress, CLU is released from the secretory pathway into the cytosol where it acts to promote autophagy, presumably making an important contribution to cellular proteostasis^8,9^. Despite these observations, and broad interest in CLU for its roles in proteostasis and cancer^4^, very little is known about the structure–function relationships of this important chaperone.

CLU undergoes complex post-translational modifications before being, under normal conditions, secreted from the cell^10^. It is translated with a 22-mer ER signal sequence which is cleaved once the protein enters the ER to generate a 427 amino acid polypeptide with a predicted mass of 50,062 Da. The protein is subsequently internally cleaved in the Golgi to generate the α- and β-chains which are joined by 5 disulfide bonds^11^ to form an anti-parallel heterodimer. The protein is also heavily N-glycosylated at six sites, to give 17–27% carbohydrate by mass; the protein has an apparent mass of 75–80 kDa in SDS PAGE, although the actual mass is ~ 58–63 kDa^12^.

This complex post-translational processing together with its propensity to form heterogenous oligomers in solution^1^ have frustrated all previous attempts to determine its three-dimensional structure. There is no empirically-determined structure for CLU, only very limited analyses by mass spectrometry^12,13^ and NMR^14^, and predictions based on amino acid sequence analyses^15,16^. Critically, the identity of specific regions of CLU that mediate its interactions with chaperone client proteins, cell receptors and other important biological ligands are completely unknown. A large part of the explanation for this lack of progress in the 35 years since CLU was discovered has been the lack of a tractable platform to manipulate its structure by mutations and efficiently express/purify the wild type protein and mutants for study.

Combined with its complex structure, the potent binding of CLU to misfolding proteins in cell culture media to form very stable high molecular weight complexes^17^, have made this an extremely challenging molecule to express as a functional recombinant protein. We and others have previously attempted expression of *r*CLU in bacteria, yeast and insect cells but in all these cases the product was structurally compromised^17–19^. Other reports of *r*CLU expression in *E. coli* and various mammalian cell lines provided little characterisation of the product and, critically, did not even test whether the product had chaperone activity^20–23^. We previously described a static-cultured HEK293 expression system that provided the first validated system to generate post-translationally processed and chaperone-active *r*CLU^17^. However this system had limitations: many large static culture flasks were required and the associated manual manipulations were laborious, including multiple chromatography steps to purify the product (and the use of a custom-made monoclonal antibody column). Collectively all these activities required an investment of several weeks of effort to generate a modest yield of purified *r*CLU (~ 4.5 mg/L of culture)^17^. Consequently, the use of this original expression system to generate large panels of CLU mutants would be extremely demanding in time and resources.

In the current work our primary aim was to remove the previous roadblock to structure–function studies of this key chaperone molecule by developing a high-throughput expression/ purification platform capable of rapidly and efficiently generating both wild type CLU and large panels of CLU mutants. This would require improvements in both speed and yield, as well as the identification of a single epitope tag system to enable the rapid uniform purification of many different mutants, while avoiding any interference with the gross structure or function of the molecule. For an extensively post-translationally modified heterodimeric protein, this was not trivial. From this starting point we trialled different cell types, culture media and conditions, media additives, transfection reagents, expression plasmids, and epitope tags and their placements, to develop an optimized work flow for the efficient generation of *r*CLU and mutants. This new optimized system is substantially faster and less laborious. Taking only ~ 1 week, and using only one shaking culture flask and a single chromatography step, this new system can achieve an order of magnitude increase in the yield of highly purified rCLU while retaining correct post-translational processing and full chaperone function. Furthermore, as a proof-of-principle, we also show how this system was used to quickly identify a rCLU mutant with impaired chaperone function. The developments we report here open up entirely new possibilities to interrogate the structure–function relationships of this long mysterious molecule.

## Results

### Optimisation of culture conditions for expression of recombinant clusterin (rCLU)

**Figure 1:**
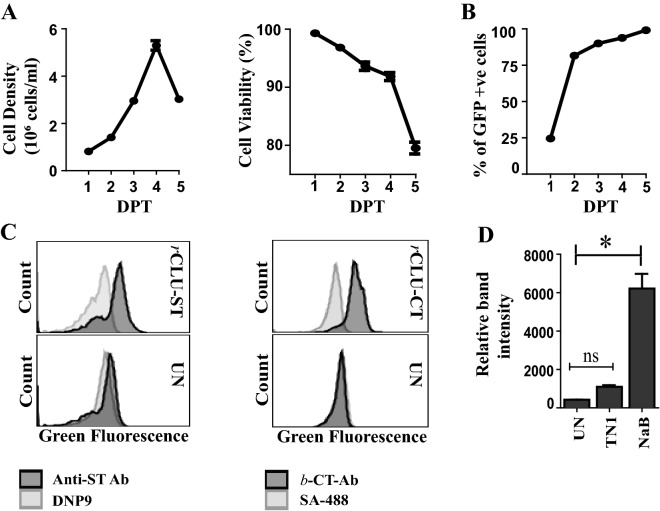
Optimisation of culture conditions and *r*CLU expression in MEXi 293E cells. (**A**) and (**B**) Data shown for MEXi293E cells transfected with pEGFP-N1; means + /− SEM (n = 3) are plotted. In some cases the error bars are too small to be visible. (**A**) *Left Panel:* Viable cell density over 5 days post transfection (DPT). *Right Panel:* Percent cell viability over 5 DPT. (**B**) Percent of cells transiently expressing GFP measured for 5 DPT. (**C**) Transfection efficiency of cells overexpressing *r*CLU-αC-ST and *r*CLU-CT were measured by immunostaining of fixed, permeabilised cells on day 5 post-transfection. Untransfected (UN) cells were used as control. To detect *r*CLU-αC-ST, cells were stained with anti-strep tag antibody (Anti-ST Ab) or DNP9 (an isotype-matched antibody of irrelevant specificity) followed by goat anti-mouse IgG-CF488. *r*CLU-CT was detected using biotinylated anti-C tag conjugate (*b*-CT-Ab) followed by streptavidin-CF488 (SA-488); the negative control in this case was cells incubated with SA-488 alone. (**D**) Densitometric analysis of Western blot with culture supernatant from cells overexpressing *r*CLU-αC-ST harvested on day 5 DPT and probed with streptactin-HRP conjugate. Cells were either supplemented with tryptone N1 (TN1) or sodium butyrate (NaB) or untreated (UN). Means + /− SEM (n = 3) are plotted; statistically significant differences are indicated by * (Oneway ANOVA, *p* < 0.001; *ns* non-significant). The results shown are each representative of two independent experiments.

**Figure 2:**
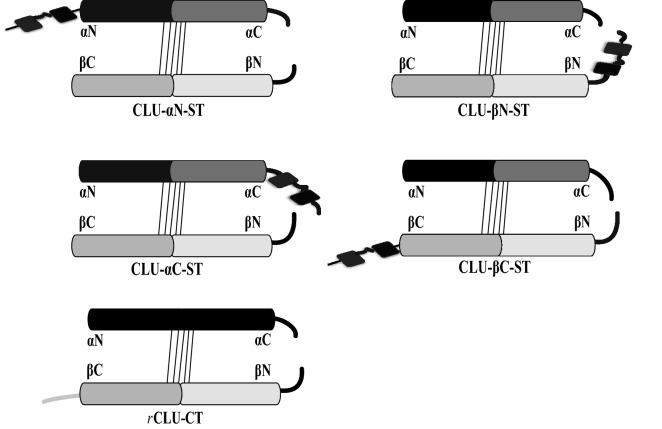
Schematic representation of affinity tag (s) placed at different positions in the mature human CLU amino acid sequence. The upper four images represent a series of constructs in which the twin Streptag sequence (ST, shown as two black rectanges joined in tandem) was positioned at the N- or C-terminus of each of the CLU α- and β-chains (i.e. αN, αC, βN or βC). The lower image represents a construct in which the 4-residue C-tag (CT, shown as a grey line) was placed at the β chain C-terminus (βC). The slanted thin black lines joining the chains represent the disulphide bonds. The thicker black lines at the right of the images represent the sequences adjoining the inter-chain cleavage site.

To assess the behaviour of transfected MEXi293E cells in culture, cells transfected with pEGFP-N1 were grown in shaking culture suspension for 4 days until they reached a density of ~ 5–6 × 10^6^ viable cells/ml (Fig. 1A). The cell viability declined slowly over days 1–4, decreasing from close to 100% at day 1 to ~ 92% at day 4; the cell viability then decreased more significantly to ~ 80% on day 5 (Fig. 1B). The proportion of viable cells expressing GFP was in the range 90–95% at 4–5 days post transfection (Fig. 1C). To further optimise culture conditions for the expression of CLU, MEXi293E cells were transfected with expression plasmids encoding WT CLU incorporating a twin Streptag at the C-terminus of the α-chain (*r*CLU-ST) or the C-tag at the C-terminus of the β-chain (*r*CLU-CT, see Fig. 2). The expression of these two proteins by transfected MEXi293E cells was measured on day 5 post-transfection by flow cytometric analysis of immunostained, fixed and permeabilised cells. These analyses estimated that typically between 70–80 and 80–90%, respectively, of transfected cells expressed these proteins (Fig. 1C). Cultures were harvested when cell viability declined to ~ 75% (~ 5–6 days after transfection), and cells removed by centrifugation at 12,000×*g* for 10 min. We also tested the effects of two widely used culture medium supplements (tryptone N1 and sodium butyrate) on the expression of *r*CLU-ST (Fig. 1D). Tryptone N1 is a casein hydrolysate which is known to promote cell growth and increase the expression of transgenes^24^ while sodium butyrate is a histone deacetylase inhibitor known to influence cell cycle progression^25^ and increase the efficiency of glucose utilization^26^. Although both supplements increased the expression of *r*CLU-ST detected by Western blotting of culture supernatants, sodium butyrate gave the largest (~ tenfold) increase (Fig. 1D).

### Optimisation of affinity tag placement in wild type recombinant clusterin

**Figure 3:**
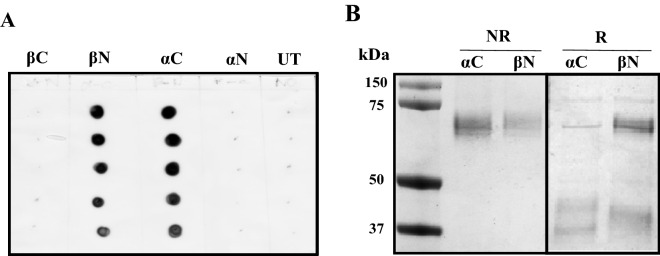
Expression and purification of *r*CLU-ST. MEXi293E cells were transfected with expression plasmids encoding each of the four *r*CLU-ST constructs (see Fig. 2) and culture supernatants were harvested on day 5 post transfection. (**A**) Image of ECL-developed immuno dot blot. Vertical columns (from top to bottom) were loaded with 12, 8, 6, 4 and 2 μl of culture supernatant. The position of the ST tag is indicated above the columns. UT represents culture supernatant from untransfected cells. (**B**) SDS PAGE of purified *r*CLU-αC-ST and *r*CLU-βN-ST. The molecular mass of protein standards (left lane) are shown in kDa. Non-reduced (NR) and reduced (R) lanes are indicated above the image; the location of the ST tag is indicated above individual lanes. The results shown are each representative of two independent experiments. The black bordering rectangles surround images of discrete parts of a single gel (original gel image provided in Supplementary Materials Figure S3A).

Using the culture conditions optimised above, we next compared the expression of a range of affinity-tagged WT *r*CLU constructs. This included those incorporating a twin Streptag sequence at four locations (at the N- and C-termini of the α- and β-chains), and a 4-residue C-tag at the C-terminus of the β-chain (Fig. 2, Supplementary Materials S1). The C-tag must always be positioned at the C-terminus of a polypeptide to be functional^27^, and the absolute C-terminus of the α-chain was not suitable because of potential interference with the cleavage motif required by the furin-like protease that cleaves the immature protein in the late Golgi compartment to form the separate α- and β-chains^28^. In dot blot analyses, *r*CLU secreted into the culture medium was detected when the twin Streptag was positioned at the α-chain C-terminus (*r*CLU-αC-ST) or at the β-chain N-terminus (*r*CLU-βN-ST), but not when the tag was positioned at either the α-chain N-terminus or the β-chain C-terminus (Fig. 3A). The expression and secretion of *r*CLU-CT was confirmed in separate dot blot assays (Supplementary Materials S2).

### Purification and structural analyses of rCLU

**Figure 4:**
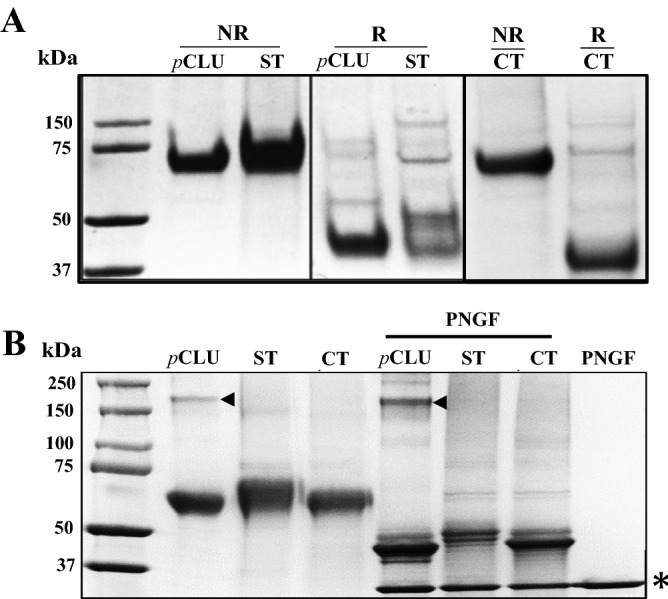
SDS PAGE analyses of *p*CLU and *r*CLU. SDS PAGE analysis of (**A**) purified *p*CLU, *r*CLU-αC-ST (ST) and *r*CLU-CT (CT) under non-reducing (NR) and reducing conditions (R), and (**B**) the same samples (non-reduced) treated with or without PNGaseF (PNGF). In (**A**) and (**B**), the molecular mass of protein standards (left lane) are shown in kDa. In (**B**), the position of a low level of contaminating human IgG that co-purifies with *p*CLU (visible on this gel) is indicated by black arrowheads, and the position of PNGaseF enzyme is indicated by an asterisk at the bottom right of the image. The very minor bands that under reducing conditions migrate to ~ 75 kDa or greater are likely to represent different glycoforms of a small fraction of *p*CLU/*r*CLU that is uncleaved when secreted. The results shown are each representative of two independent experiments. In (**A**), the black bordering rectangles surround images of discrete parts of gels. The complete gel images corresponding to the left and centre panels in (**A**), and the right panel, are shown in Supplementary Materials Figures S3B and S3C, respectively.

*r*CLU-αC-ST, *r*CLU-βN-ST and *r*CLU-CT were purified by affinity chromatography as described in Materials and Methods. SDS PAGE analysis of *r*CLU-αC-ST and *r*CLU-βN-ST showed that under non-reducing conditions both migrated to a position corresponding to ~ 70 kDa, similar to CLU purified from human plasma (*p*CLU) (Figs. 3B, 4A). However, under reducing conditions, ~ 60–70% of *r*CLU-βN-ST did not dissociate into faster-migrating α- and β-chains, but rather still migrated at ~ 70–75 kDa; the remaining 30–40% of the protein migrated to a position at ~ 40–45 kDa. This suggested that *r*CLU-βN-ST had been incompletely cleaved during its processing in the Golgi. In contrast, under reducing conditions, ~ 90–95% of *r*CLU- αC-ST dissociated and migrated to form two bands positioned between ~ 40 and 46 kDa. The position of these bands is consistent with the faster migrating one representing the untagged β-chain, and the upper more slowly migrating band representing the α-chain bearing at its C-terminus the 38-residue (~ 3.5 kDa) twin Streptag (Fig. 3B). Further SDS PAGE analyses showed that, under non-reducing conditions, *p*CLU, *r*CLU-αC-ST and *r*CLU-CT migrated to similar positions (at ~ 70–75 kDa; Fig. 4A). Under reducing conditions, the α- and β-chains of *p*CLU co-migrate as expected^3^, the Streptag-bearing α-chain of *r*CLU-αC-ST migrated more slowly than the untagged β-chain (as also seen in Fig. 3B), and the α- and β-chains of *r*CLU-CT do not resolve, like *p*CLU (Fig. 4A). The slightly faster migration of *r*CLU-CT relative to *p*CLU, most apparent under reducing conditions, may relate to small differences in glycosylation between the two proteins. Consistent with this suggestion, when most of the glycosylation was enzymatically removed from these molecules by digestion with PNGase^13^, they migrated to similar positions in reducing SDS PAGE; similarly treated *r*CLU-αC-ST migrated slightly more slowly, to a position consistent with its additional ~ 3.5 kDa twin Streptag (Fig. 4B).

### Purification yields and structural analyses of rCLU

**Figure 5:**
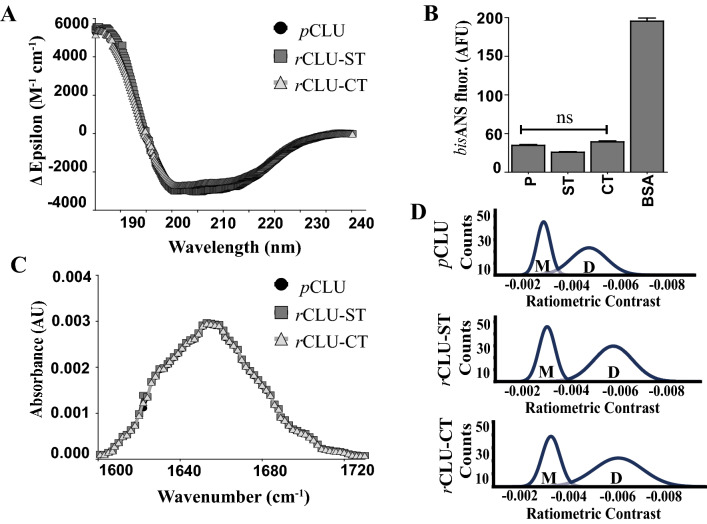
Biophysical characterisation of *r*CLU using Far-UV CD, FT-IR, *bis*ANS and mass photometry. (**A**) Far-UV CD spectra of *p*CLU, *r*CLU-ST (*r*CLU-αC-ST) and *r*CLU-CT. Means + /− SEM (n = 10) are plotted, error bars too small to be visible. (**B**) *bis*ANS analysis of *p*CLU (P), *r*CLU-αC-ST (ST), *r*CLU-CT (CT) and BSA. Mean fluorescence values (AFU, arbitary fluorescence units) + /− SEM (n = 3) are plotted. There was no significant differences (ns) between the *bis*ANS fluorescence for any of the CLU proteins (Oneway ANOVA, *p* > 0.05). (**C**) FT-IR spectra for *p*CLU, *r*CLU-αC-ST and *r*CLU-CT. Mean absorbances (AU, arbitary absorbance units) + /− SEM (n = 3) are plotted. (**D**) Mass photometry plots showing the distribution of putative monomer (M) and dimer (D) forms of *p*CLU, *r*CLU(-αC)-ST and *r*CLU-CT at 1 nM in PBS. The results shown are each representative of three independent experiments.

The yields obtained for *r*CLU-αC-ST and *r*CLU-CT were similar and in the range of 20–50 mg/L (Table 1). This is approaching an order of magnitude increase in yield from the previously published method which expressed *r*CLU in adherent HEK293 cells grown in static culture flasks^17^. The purity of the *r*CLU products obtained using the single-step affinity chromatography protocols was excellent in both cases (Fig. 4A,B). Far-UV circular dichroism (CD) analyses of purified *p*CLU, *r*CLU-αC-ST and *r*CLU-CT gave very similar results in all cases (Fig. 5A), with only minor differences in the content of α-helix and β-strand structures predicted for each protein (Table 2). Using bisANS fluorescence assays to compare the levels of hydrophobicity exposed on each of these three proteins indicated very little difference between them, all being much less than the hydrophobic control protein BSA (Fig. 5B). Further analysis of the same three proteins by FT-IR, to compare overall secondary structure content and conformation^29^, also revealed no significant differences (Fig. 5C).

**Table 1:** Comparison of yield, structure and chaperone activity of purified *r*CLU.

CLU type	Total no. of amino acid residues	No. of residues in tag	Tag as % of native CLU sequence	Yield (mg/L)	SDS-PAGE analysis, chaperone activity, Far-UV CD, FT-IR, *bis*ANS, mass photometry
*r*CLU-αC-ST	465	38	~ 8	~ 20–30	Similar to *p*CLU
*r*CLU-CT	431	4	~ 0.9	~ 30–50	Similar to *p*CLU

**Table 2:** Secondary structure content of *p*CLU and *r*CLU predicted from Far-UV CD data.

Sample	α-helix	β-strand	Turn	Disordered
*p*CLU	10.37	83.51	1.35	4.77
*r*CLU-αC-ST	12.05	81.78	1.36	4.81
*r*CLU-CT	12.14	81.81	1.35	4.69
*r*CLU^Δ17–47^-αC-ST	10.65	83.08	1.44	4.83

Lastly, we took advantage of access to a new technology, mass photometry^30^, to compare the distribution of *r*CLU-αC-ST, *r*CLU-CT and *p*CLU between different oligomeric species in solution. When tested at a 1 nM concentration in PBS (pH 7.4), the relative distributions of species between putative monomer (i.e. a single disulphide-linked α-β structure) and dimer (oligomer comprised of two non-covalently associated α-β chain structures) was similar for all three proteins; the putative dimer comprised ~ 60% of the species in solution with the remainder being putative monomer (Fig. 5D). The ratiometric contrast values for the dimer population of *p*CLU appear, however, slightly less than those for *r*CLU-αC-ST and *r*CLU-CT. Ratiometric contrast can be affected by both refractive index and mass. The *r*CLU molecules have been expressed by a cell line, while *p*CLU has been secreted by multiple tissues into the blood; it is therefore expected that glycosylation will be similar between the different *r*CLU molecules but different to *p*CLU. It is likely that this difference in glycosylation affects refractive index and/or mass sufficiently to produce the observed small difference in ratiometric contrast.

### Assessing the in vitro chaperone activity of rCLU

**Figure 6:**
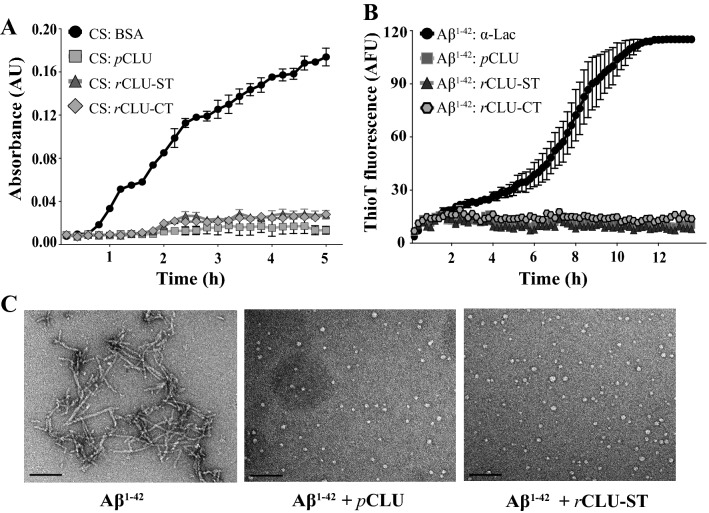
In vitro comparison of chaperone activities of *p*CLU and *r*CLU. (**A**) Amorphous aggregation of CS. CS (2 µM) was incubated with or without 2 µM of *p*CLU, *r*CLU(-αC)-ST, *r*CLU-CT or BSA (used as a non-chaperone control protein). Mean absorbances at 360 nm + /− SEM (n = 4) are plotted. (**B**) Amyloid-β aggregation: Aβ^1–42^ (10 µM), supplemented with 20 μM ThioT, was incubated with 1 μM α-lactalbumin (a non-chaperone control protein) or with 1 μM of *p*CLU, *r*CLU(-αC)-ST, or *r*CLU-CT for 14 h*.* Mean ThioT fluorescence values (AFU, arbitary fluorescence units) + /− SEM (n = 4) are plotted. (**C**) TEM images of samples taken at 12 h from Aβ^1–42^ aggregation reactions incubated with or without *p*CLU and *r*CLU-αC-ST at a molar ratio of Aβ^1–42^: CLU = 10:1. Scale bar represents 100 nm. The results shown are each representative of three independent experiments.

The abilities of *r*CLU-αC-ST, *r*CLU-CT and *p*CLU to inhibit amorphous and amyloid protein aggregation in vitro were compared. In the CS aggregation assays, the addition of the non-chaperone control protein BSA had little effect on the time-dependent increase in turbidity measured, however *r*CLU-αC-ST, *r*CLU-CT and *p*CLU all provided near-complete inhibition of CS aggregation under the conditions tested (Fig. 6A). A similar result was obtained when testing the effects of these three proteins on the in vitro aggregation of Aβ^1–42^. The non-chaperone control protein α-lactalbumin had little effect on the time-dependent increase in Thio-T fluorescence associated with Aβ^1–42^ amyloid formation while *r*CLU-αC-ST, *r*CLU-CT and *p*CLU all essentially completely inhibited this (Fig. 6B). The ability of *p*CLU and *r*CLU-αC-ST to inhibit Aβ^1–42^ amyloid fibril formation was further confirmed by TEM analysis of samples taken at 12 h from the Aβ^1–42^ aggregation reactions. Both *p*CLU and *r*CLU-αC-ST completely inhibited the formation of visible fibrils and only small spherical aggregates were detected in the presence of CLU, as reported previously (Fig. 6C)^31^.

### Production and structural testing of a rCLU mutant

**Figure 7:**
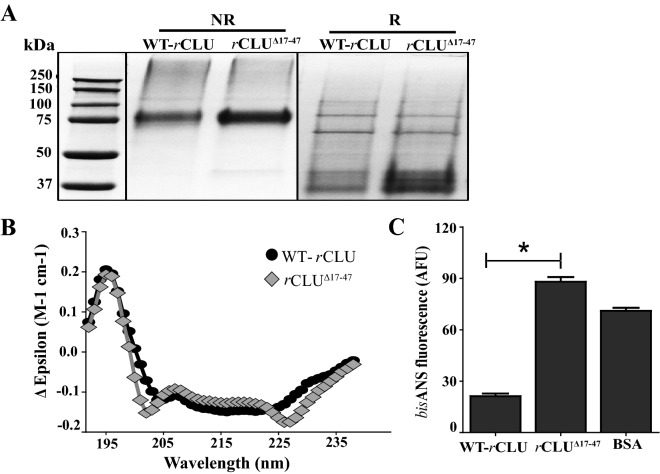
Structural analyses of wild type and mutant *r*CLU proteins. Full length WT-*r*CLU-αC-ST (WT-*r*CLU) and a CLU deletion mutant *r*CLU^Δ17–47^-αC-ST (*r*CLU^Δ17–47^, in which residues 17–47 of the mature protein were deleted) were analysed by SDS PAGE, Far-UV CD and *bis*ANS assay. (**A**) SDS PAGE. Samples were analysed both under non-reducing (NR) and reducing conditions (R). The molecular mass of protein standards (left lane) are shown in kDa. The black bordering rectangles surround images of discrete parts of gels (original corresponding gel images provided in Supplementary Materials Figure S3D and S3E). (**B**) Far-UV CD spectra of WT-*r*CLU and *r*CLU^Δ17–47^. Means + /− SEM (n = 10) are plotted; error bars are too small to be visible. See Table 2 for predicted structure contents. (**C**) *bis*ANS analysis of WT-*r*CLU, *r*CLU^Δ17–47^ and BSA. Mean values of *bis*ANS fluorescence (AFU, arbitrary fluorescence units) + /− SEM (n = 3) are plotted. The *bis*ANS fluorescence of *r*CLU^Δ17–47^ was significantly greater than that of WT-*r*CLU (indicated by *, Oneway ANOVA, *p* < 0.001). The results shown are each representative of three independent experiments.

Prior to this report, the substantial technical difficulties associated with the expression and purification of chaperone-active *r*CLU severely limited any attempts to study CLU mutants. In fact, to our knowledge, no CLU mutant has ever before been expressed and purified to allow in vitro testing of its properties. The ability to generate CLU mutants is essential to open the gateway for an interrogation of the structure–function relationships of this key chaperone molecule. It was also previously largely unknown how manipulations of the CLU sequence might impact on its processing and secretion from the cell. As a proof of principle, we tested whether it would be possible to use the *r*CLU expression/purification platform we developed to rapidly generate CLU mutants in which selected stretches of CLU sequence were deleted. We initially expressed and purified *r*CLU^Δ17–47^-αC-ST (which contains a deletion towards the N-terminus of the α-chain; Supplementary Materials S1), and then subjected the purified mutant protein to analysis by SDS PAGE, Far-UV CD, and bisANS analyses. In non-reducing SDS PAGE, WT- and *r*CLU^Δ17–47^-αC-ST migrated to similar positions at ~ 75 kDa, and under reducing positions two major bands corresponding to the α-chain (bearing a twin Streptag) and the β-chain the were detected at ~ 37–40 kDa (Fig. 7A). The separation between the bands corresponding to the α- and β-chains is slightly less in the case of *r*CLU^Δ17–47^-αC-ST, presumably as a result of the small deletion from the α-chain.

Far-UV circular dichroism (CD) showed similar patterns for WT- and *r*CLU^Δ17–47^-αC-ST with some minor differences observed at ~ 202 nm and ~ 230 nm (Fig. 7B). These small differences relative to WT-*r*CLU-αC-ST corresponded to predictions of a 2% increase in the β-strand content and a 2% decrease in α-helix content (Table 2). In contrast, *r*CLU^Δ17–47^-αC-ST had a significantly increased level of exposed hydrophobicity relative to WT-*r*CLU-αC-ST, as assessed by bisANS fluorescence (Fig. 7C).

## Discussion

Several important lessons taken from previous attempts to generate recombinant CLU can be summarised as follows. Firstly, a mammalian expression system is essential to obtain correctly post-translationally modified and chaperone-active *r*CLU. Secondly, using current technologies, shaking cultures of mammalian cells are needed to obtain maximum cell densities and hence the greatest yields. Under these conditions, culture media proteins are subjected to sustained elevated temperature (37 °C) and shear stress, conditions that induce progressive misfolding of some media proteins. Therefore, thirdly, to avoid low *r*CLU yields and complicated purification requirements arising from the irreversible binding of the potently chaperone-active *r*CLU to misfolded culture media proteins, it is essential to grow the cells in protein-free media.

In recent years, genetically engineered human cell lines derived from human embryonic kidney cells (HEK293) have become commercially available that are capable of growing in shaking culture, in specialist protein-free media, to extraordinarily high densities of ~ 5–15 × 10^6^ cells/ml. These cells are easily transfected and have been engineered to enable them to episomally replicate plasmids. This has the practical consequence that during the short term rapid growth of transiently transfected cells, the level of recombinant protein expression is maximized. This was confirmed in preliminary work with cells transfected to express soluble enhanced green fluorescent protein (GFP) and cultured as described (Fig. 1B). We exploited the features of one of these new mammalian expression systems (MEXi293E cells) to develop a near-ideal platform for the bulk expression of chaperone-active *r*CLU. In addition, as outlined in the Introduction, a primary goal was to develop a high-throughput expression/purification platform suitable for the rapid and efficient generation of large panels of *r*CLU mutants. To achieve this required us to develop the ability to rapidly purify many different CLU mutants using a uniform single-step affinity chromatography protocol. This in turn necessitated identification of a suitable epitope tag, and critically its placement in the molecule, compatible with these demands while avoiding the tag itself significantly impacting on the structure or function of CLU. For an extensively post-translationally modified heterodimeric protein, this was not trivial.

Excluding internally-placed tags, there are four possible “terminal” positions for an epitope tag in the heterodimeric structure of CLU but no previous knowledge of which if any of these placements might interfere with post-translational processing or function. In order to identify an “ideal” epitope tag system, we first tested the twin Streptag at all 4 terminal placements and also the C-tag placed at the C-terminus of the b-chain (Fig. 2). When the twin Streptag sequence was placed at either the α-chain N-terminus or the β-chain C-terminus, secreted *r*CLU was not detected in the culture medium (Fig. 3A), suggesting that when in those positions the tag was interfering with the correct processing and/or secretion of the protein. In contrast, when the twin Streptag was located at the α-chain C-terminus (separated from the inter-chain cleavage motif by a 4-glycine spacer) or at the β-chain C-terminus, the corresponding *r*CLU proteins were robustly detected in the culture medium (Fig. 3A). However, SDS PAGE analysis indicated that the *r*CLU-βN-ST variant displayed impaired proteolytic processing into α- and β-chains (Fig. 3B). Thus, of the four locations for the twin Streptag tested (Fig. 2), the *r*CLU-αC-ST variant proved to be the one that behaved most ideally, and the purified protein was very similar in both structure and chaperone function to *p*CLU (Figs. 4, 5, 6). The only real potential disadvantage to this construct is the size of the twin Streptag sequence, which in the form used is comprised of 38 residues and an approximate molecular weight of 3.5 kDa (i.e. ~ 7% of the total mass of the mature CLU polypeptide). In an attempt to address this, we tested single Streptag versions of *r*CLU, but found that the purification of this using Streptactin XT was significantly less efficient (data not shown). In later work we also tested the very short 4-residue C-tag placed at the β-chain C-terminus, which provided good expression and similar yields to the *r*CLU-αC-ST construct. Either tag provided the capacity to rapidly affinity purify *r*CLU under non-denaturing conditions in a single-step to very high purity (Fig. 4A). Compared with *p*CLU, MEXi293E cell expressed WT *r*CLU (-αC-ST or -CT) is correctly cleaved into α- and β-chains, is similarly glycosylated, has similar structural features to *p*CLU, and shows a near-identical chaperone activity (Figs. 4, 5, 6; Table 1). Of the feasible affinity tag options we have identified in this study, the C-tag appears ideally suited to the job of allowing rapid purification of correctly processed *r*CLU molecules with minimal modification to the native sequence.

Importantly, we have also demonstrated the feasibility of using the new expression/purification platform to generate and test the structure and function of designed CLU mutants (Fig. 7). To demonstrate feasibility, we have shown preliminary results for the expression, purification and limited structural testing of a deletion mutant *r*CLU^Δ17–47^. The region containing CLU residues 17–47 was chosen because this sequence is highly conserved between different species (67% identity and 82% similarity between human, rabbit, rat and dog) and residues 23–34 within this sequence are predicted to be a region of structural disorder^32^. These features implicated this sequence as being one that, if deleted, could significantly impact on the overall structure and post-translational processing of CLU, making it a challenging molecule to express and purify. Although SDS PAGE and far UV CD analysis did not detect any substantial changes in post-translational processing or overall secondary structure content, a significant increase in *bis*ANS fluorescence suggested that a conformational change resulting from the deletion had produced region(s) of greater surface exposed hydrophobicity (Fig. 7C). Mutated molecules are well known to often express at reduced levels compared to the parent wild type molecule. We obtained a yield for *r*CLU^Δ17–47^ of ~ 2.0 mg/L, which is about tenfold lower than that obtained for *r*CLU-αC-ST (Table 1), but thanks to the inherently high level of expression that can be achieved using this system the yield was more than sufficient for multiple subsequent analyses. This suggests that the expression/purification platform is very likely to enable the generation of panels of CLU mutants, including those that may feature some structural alterations. We expect that extension of this mutagenesis approach will in time allow us to identify specific CLU regions that interact with chaperone client proteins, specific cell receptors and other biologically important ligands.

In summary, this report describes a rapid and efficient method to produce structurally and functionally validated *r*CLU that now enables a range of important applications that were previously difficult or impossible: (1) For the first time, express and characterise panels of CLU mutants, to identify specific regions important in the structure and function of this important chaperone. (2) Produce large quantities of *r*CLU for use in structural studies, or as a therapeutic agent to treat a wide range of protein deposition diseases or other conditions. (3) Site-specifically label CLU (e.g. for single molecule fluorescence studies). (4) Metabolically label CLU for NMR or other biophysical analysis. The potent chaperone activity of CLU is thought to exert a central and critical physiological role by neutralizing the toxicity of misfolded proteins and facilitating their clearance from the body. Thus, the advances described in this report open the door to greater understanding of how CLU performs this and other important physiological roles. Furthermore, it gives us the ability to potentially develop this new knowledge towards improvements in human health.

## Methods

### Materials

4,4′-Dianilino-1,1′-binaphthyl-5,5′-disulfonic acid (bisANS) dipotassium salt, alpha-lactalbumin, bovine serum albumin (BSA), citrate synthase, 2-mercaptoethanol, sodium butyrate, thioflavin T (ThioT) and Greiner 384-well clear flat bottom plates were purchased from Sigma Aldrich (Australia). Pluronic F-68 (PF-68) was purchased from Thermo Fisher Scientific (Australia). Human beta-amyloid (1–42) was purchased from AnaSpec (Australia). Tryptone N1 was a kind gift from Organotechnie (France). All other chemicals were of analytical grade and purchased from Sigma Aldrich (Australia) unless otherwise specified.

### Plasmids

A series of plasmids encoding wild type human CLU (WT CLU) were designed to screen for an optimal affinity tag type and tag position. DNA sequences encoding a tandem version of strep tag II known as twin-strep tag with an internal linker sequence (WSHPQFEK-*GGGSGGGSGGS*-*SA*WSHPQFEK; ST), or EPEA (C-tag; CT) were incorporated into the CLU cDNA sequence at various positions (Fig. 2, Supplementary Materials S1). These sequences were synthesized as fragments by Gene Universal Inc. (USA) and cloned into pcDNA3.1 ( +) or pDEST40 vectors (Thermo Fisher Scientific, Australia). Mutant CLU sequences, in which deletions of variable length were made, were produced in the same way and cloned into pcDNA3.1 ( +) for expression. pEGFP-N1 (encoding EGFP; Addgene No. 6085–1) was used to monitor the transfection efficiency and transgene expression in cells over a period of 5–6 days. Plasmids for transfection were purified using the Compact Prep Kit purchased from Qiagen (Australia).

### Cell culture and transfection

MEXi293E (IBA Life Sciences) cells were grown in MEXi 293E cultivation media. Cells were seeded at a density of 0.5–1 × 10^6^ cells/ml into media and grown in an Eppendorf S41i shaker-incubator at 37 °C, with 5% (v/v) CO_2_ in air and shaking at 120 rpm. Cell density and viability were assessed as required using trypan blue staining and a hemocytometer, and cells were passaged at a density of 3–3.5 × 10^6^ cells/ml. In preparation for transfection, MEXi 293E were grown in 250 ml vented Corning Erlenmeyer shaker flasks (Sigma Aldrich, Australia) to a cell density of 2–3 × 10^6^ cells/ml, and a cell viability of at least 98%. Prior to transfection MEXi 293E cells were resuspended in fresh transfection medium (IBA Life Sciences) at 1–1.5 × 10^6^ cells/ml. The cell suspension was pipetted up and down (gently) 2–3 times before adding to a 250 ml shaker flask. Plasmid DNA at 1.5 mg/L of culture volume was added to the flask and incubated for 10 min in the cell culture-shaker incubator. Linear polyethylenimine, transfection grade (PEI 25 K, Polysciences, Inc. USA) was then added at 7.5 mg/L of culture volume, and mixed immediately by swirling the flask and placing it in the cell culture-shaker incubator. Cells were supplemented with 0.1% v/v PF-68 at 24 h post transfection and 2 mM sodium butyrate at 48 h post transfection. Cultures were harvested when cells reached a density of 4–5 × 10^6^ cells/ml and when the cell viability had dropped to ~ 75% (day 5 or day 6). To harvest, cultures were centrifuged at 4 °C, first at 1,200×*g* for 5 min at (to remove cells and large debris) and then at 12,000×*g* for 10–15 min (to remove fine particulates).

### Purification of human plasma CLU and rCLU

Human plasma CLU (*p*CLU) was purified by immunoaffinity chromatography of plasma prepared from human blood as described before^33^. *r*CLU-αC-ST was purified using a 5 ml Streptactin-XT Superflow high capacity cartridge (IBA Life Sciences); in these cases, before purification, the culture supernatant was dialysed × 3 against phosphate buffered saline (PBS: 137 mM NaCl, 2.7 mM KCl, 7.9 mM Na_2_HPO_4_, 1.5 mM KH_2_PO_4_, pH 7.4) to remove any biotin present in the media. *r*CLU-CT was purified using a 5 ml Capture Select C-tag column (Thermo Fisher Scientific, Australia). Purifications were performed following the manufacturer’s instructions; protein bound to the C-tag column was eluted with 2 M MgCl_2_ in 20 mM Tris buffer, pH 8.0. Affinity eluates were dialysed × 3 against PBS and concentrated using Amicon Ultra15 centrifugal concentrators (Merck Millipore Australia). The concentration of purified proteins was estimated by spectrophotometry (A^280^) and they were stored in PBS at − 20 °C until further use.

### Immunostaining and flow cytometry

Transfection efficiency for transfected cells expressing *r*CLU was measured by immunostaining of fixed and permeabilized MEXi 293E cells harvested at day 5 post-transfection. Briefly, cells were washed with PBS, fixed for 10 min on ice with 4% w/v paraformaldehyde in PBS (PFA) solution and permeabilised for 15 min on ice with 0.5% v/v Triton-X 100 in PBS. Subsequently, cells were blocked with 1% w/v BSA in PBS (BSA/PBS) and *r*CLU-αC-ST expression detected using anti-strep tag monoclonal antibody raised in mouse (anti-ST-Ab, IBA Life Sciences, diluted 1:200 in BSA/PBS); DNP9^3^, an antibody of irrelevant specificity, was used as an isotype-matched control. Immunostaining of cells overexpressing *r*CLU-CT was done using biotinylated anti-C tag conjugate (*b*-CT-Ab, Thermo Fisher Scientific Australia, diluted 1:200 in BSA/PBS). Goat anti-mouse-IgG CF488 (anti-mIgG-488, Biotium Australia) and Streptavidin-CF488 (ST-488, Biotium Australia), both diluted 1:400 in BSA/PBS, were used as secondary detection reagents for *r*CLU-αC-ST and *r*CLU-CT, respectively. Immunostained cells, cells transfected with pEGFP-N1 plasmid, and untransfected cells, were analysed by flow cytometry using a Becton Dickinson LSRII Fortessa X-20 (BD Biosciences, USA) and BDFACSDiVa Software (BD Biosciences, USA). Excitation was at 488 nm, and the fluorescence of cells overexpressing EGFP, or immunostained to detect *r*CLU expression, was collected using a 525/25 nm band pass filter. When analysing live cells for EGFP fluorescence, immediately prior to acquisition samples were supplemented with 1 μg/ml propidium iodide (PI) and the PI emission was collected using a 696/40 nm band pass filter; PI-stained dead cells were electronically excluded from the analyses. For each sample, approximately 10,000 events were collected; data acquired was later analysed using FlowJo software v 3.1 (BD Biosciences, USA).

### SDS PAGE, native gel electrophoresis and immunoblotting

SDS PAGE was performed using 4–12% Bolt Bis–Tris gels and 1 × MES-SDS running buffer (Thermo Fisher Scientific, Australia). Proteins were diluted in sample buffer (60 mM Tris pH 6.8, 1% (w/v) SDS, 10% (v/v) glycerol, 0.01% (w/v) bromophenol blue) and heated at 95 °C for 5 min prior to loading and electrophoresis was performed at 150 V. Where required, samples were reduced with the addition of 5% v/v 2-mercaptoethanol prior to heating. Gels were stained with Coomassie Blue (0.2% w/v Coomassie Blue R250, 40% v/v methanol, 10% v/v glacial acetic acid) and destained in 40% v/v methanol, 10% v/v glacial acetic acid.

For immunoblotting, following separation by SDS PAGE, samples were electrophoretically transferred onto a nitrocellulose membrane (Pall Corporation, USA) using a Trans Blot (Bio-Rad, Australia) in Tris glycine transfer buffer (25 mM Tris–HCl, 192 mM glycine, pH 7.6, 20% v/v ethanol). The membrane was blocked for 1 h at room temperature in 3% w/v BSA in PBS (for detection of *r*CLU-αC-ST) or 5% w/v skimmed milk in PBS (SM/PBS; for detection of *r*CLU-CT or plasma CLU). To detect *r*CLU-αC-ST, the blot was probed overnight at 4 °C either with anti-strep tag monoclonal antibody (IBA Life Sciences) diluted 1:4,000 in 0.2% w/v BSA in PBS containing 0.01% v/v Tween-20, or a 1:1 mixture of undiluted G7 and 41D mouse hybridoma culture supernatant (G7 and 41D are monoclonal antibodies specific for human CLU)^3^. Bound primary antibodies were detected using goat anti-mouse IgG-HRP (Dako Agilent) diluted 1:5,000 in SM/PBS. For detection of *r*CLU-CT, the blot was probed with Capture Select biotin anti-C tag conjugate (Thermo Fisher Scientific, Australia) diluted 1:1,000 in SM/PBS followed by incubation with streptavidin-HRP conjugate (Abcam, ab7403) diluted 1:5,000 in SM/PBS. Following washing, an enhanced chemiluminiscence (ECL) kit (Supersignal Western Pico; Thermo Fisher Scientific, Australia) and Amersham 600 RGB imager (GE Healthcare, Australia) were used for chemiluminescence imaging of the blots following the manufacturer’s instructions. In immunodot blot assays, small volumes of culture supernatants (2–12 μl) were directly applied to nitrocellulose membrane and processed as described above for immunoblotting.

### In-vitro deglycosylation of CLU

5 μg of *p*CLU, *r*CLU-αC-ST or *r*CLU-CT proteins were incubated with or without 20 μg of recombinant PNGaseF diluted in 30 μl of PBS. Samples were gently agitated for 20 h at RT and subsequently analysed by non-reducing SDS PAGE. Recombinant PNGaseF was expressed in *E. coli* transformed with a plasmid encoding PNGaseF tagged at the C-terminus with 6his (popH6-PNGaseF, addgene #40,315). Expression, and purification using nickel affinity chromatography, was performed as described in^34^.

### Circular dichroism and Fourier-transformed infrared spectroscopy

Far-UV circular dichroism (CD) data was acquired using a Jasco Model J-810 spectropolarimeter connected to a CDF-426S/L Peltier system (Jasco). Purified protein samples were prepared at 0.1 mg/ml in 5 mM phosphate buffer pH 7.4 (filtered and degassed) and analysed using a 0.1 cm cuvette. 10 scans were acquired for each sample at 37 °C at a bandwidth of 1 nm, a sensitivity of 5 millidegrees, and continuous scanning mode at a speed of 100 nm/min. Data was plotted as the mean values for all scans. Far-UV (190–250 nm) CD spectra was recorded and analysed using DichroWeb software against the SMP 180 data set optimised for 190–240 nm using the CONTIN program^35,36^. For FT-IR spectroscopy, 20 μl of purified *r*CLU or *p*CLU (at 10 μM in PBS) was added into a BioATR cell. Samples were acquired in absorbance mode at 37 °C on a Vertex 70 FT-IR spectrometer (Bruker Optics, UK) and corrected for the absorption spectra of PBS.

### Mass photometry

To assess the relative distribution of monomeric and oligomeric forms of CLU, proteins were diluted to a concentration of 1 nM in PBS and 10 µl of each sample was analysed over 10 min at a rate of 600 frames/ min with a ONEMP mass photometer (Refeyn LTD, Oxford, UK). Data was acquired and analysed using AcquireMP and DiscoverMP (v1.2.3) softwares (Refeyn LTD, v1.1.3), respectively, as reported previously^37^.

### bisANS assay

To assess the degree of surface exposed hydrophobicity, proteins at 0.1 mg/ml in PBS were incubated with 20 μM bisANS for 10 min at room temperature in a Greiner 384-well plate. Subsequently, fluorescence was measured on a POLARstar Omega plate reader using 360/10 nm and 490/10 nm band pass filters for excitation and emission, respectively. Samples were analysed in triplicate and all readings were corrected for the fluorescence of bisANS in PBS.

### Protein aggregation assays

The effects of purified *p*CLU, *r*CLU-αC-ST and *r*CLU-CT molecules on both amorphous and amyloid protein aggregation were tested in vitro. Citrate synthase (CS) was used as an amorphously aggregating client protein, and amyloid-beta 1–42 (Aβ^1–42^) as an amyloid-forming client protein. CS (2 μM in 50 mM Tris, 8 mM HEPES, pH 8.0, 50 μl/well) was induced to aggregate by heating at 43 °C for 4–5 h in a Greiner flat bottom 384 clear-well plate (Sigma Aldrich, Australia). BSA were used as a non-chaperone control proteins at same concentrations as CLU. CLU and BSA were tested at final concentrations of 1 μM and 2 μM. Samples were acquired using a Spectrostar Nano plate reader (BMG Labtech, Germany) with 20 light flashes per reading and 3 s double orbital shaking between each read (1 mm shaking width, 600 rpm). Aggregation of CS was measured as changes in turbidity (A^360^). Aβ^1–42^ (10 μM in PBS supplemented with 20 μM ThioT, 50 μl/well) was incubated without shaking at 30 °C in a 384 well flat clear bottom plate (Sigma Aldrich, Australia) with or without 1 μM CLU, or 1 μM alpha-lactalbumin (used as a non-chaperone control protein). A POLARstar Optima plate reader (BMG Labtech, Germany) equipped with 440/10 nm (excitation) and 520/25 nm (emission) band pass filters, was used to measure ThioT fluorescence over 14 h.

### Transmission electron microscopy (TEM)

Samples (5 μL) taken at the 12 h time point from aggregation reactions containing Aβ^1–42^ alone, or Aβ^1–42^ mixed with *p*CLU or *r*CLU-ST, were applied to Formvar-coated copper grids. The samples were stained with 2% (w/v) uranyl acetate, and imaged using a FEI Tecnai T12 transmission electron microscope (Cryogenic Electron Microscopy Facility, Molecular Horizons, University of Wollongong). Images were analysed using the SIS Megaview II Image Capture system (Olympus).

### Statistical tests

Wherever required statistical analyses of data was performed using Oneway-ANOVA with Bonferonni comparison of all pairs with significant outcomes selected on the basis of highest confidence level (*p* < 0.001).

### Ethics

Human blood samples were collected from Wollongong Hospital with ethics approval obtained from the Human Ethics Committee at the University of Wollongong (HE02/080). All experiments were performed in accordance with the relevant guidelines and regulations. Written informed consent was obtained from all donors involved in this study, who were being treated by standard venepuncture for polycythemia or hemochromatosis.

## Supplementary information

Supplementary information.
