# Supplementary material for: Rapid high-yield expression and purification of fully post-translationally modified recombinant clusterin and mutants
Source: Sci Rep. 2020 Aug 28;10:14243. doi: 10.1038/s41598-020-70990-3 (PMC7455699; doi:10.1038/s41598-020-70990-3)
Supplement: Supplementary file 1 — Supplementary information. [file 41598_2020_70990_MOESM1_ESM.pdf]

Rapid high-yield expression and purification of fully post-translationally modified recombinant clusterin and mutants

Sandeep Satapathy[1, 2], **Rebecca A Dabbs**[3], **Mark R Wilson**[1, 2,*]

[1] Illawarra Health and Medical Research Institute, School of Chemistry and Molecular Bioscience, University of Wollongong, Wollongong, NSW, Australia. 2522.
[2] Molecular Horizons Research Institute, University of Wollongong, Wollongong, NSW. Australia. 2522.
[3] Burnett Institute, 85 Commercial Road, Melbourne, VIC. 3004. Australia

* Corresponding author. *Postal address:* Rm 313, Building 42 (Molecular Horizons), University of Wollongong. Northfields Avenue, Wollongong. NSW. 2522. Australia; *Telephone:* +61242214534; *Email:* mrw@uow.edu.au.

Supplementary Materials

Supplementary Materials S1: Full length amino acid sequences of different *r*CLU constructs (see main text Figure 2)

Key for constructs 1-4 (twin Streptag):

MMKTLLLFVGLLLTWESGQVLG: signal peptide sequence

GGGG: 4 x glycine spacer

SAWSHPQFEKGGGSGGGSGGSAWSHPQFEK: twin Streptag sequence

1. *r*CLU-αN-ST

MMKTLLLFVGLLLTWESGQVLGDQTVS***GGGG*****SAWSHPQFEKGGGSGGGSGGSAWSHPQFEK*****GGGG***DNELQEMSNQGSKYVNKEIQNAVNGVKQIKTLIEKTNEERKTLLSNLEEAKKKKEDALNETRESETKLKELPGVCNETMMALWEECKPCLKQTCMKFYARVCRSGSGLVGRQLEEFLNQSSPFYFWMNGDRIDSLLENDRQQTHMLDVMQDHFSRASSIIDELFQDRFFTREPQDTYHYLPFSLPHRRPHFFFPKSRIVRSLMPFSPYEPLNFHAMFQPFLEMIHEAQQAMDIHFHSPAFQHPPTEFIREGDDDRTVCREIRHNSTGCLRMKDQCDKCREILSVDCSTNNPSQAKLRRELDESLQVAERLTRKYNELLKSYQWKMLNTSSLLEQLNEQFNWVSRLANLTQGEDQYYLRVTTVASHTSDSDVPSGVTEVVVKLFDSDPITVTVPVEVSRKNPKFMETVAEKALQEYRKKHREE

2. *r*CLU-αC-ST

MMKTLLLFVGLLLTWESGQVLGDQTVSDNELQEMSNQGSKYVNKEIQNAVNGVKQIKTLIEKTNEERKTLLSNLEEAKKKKEDALNETRESETKLKELPGVCNETMMALWEECKPCLKQTCMKFYARVCRSGSGLVGRQLEEFLNQSSPFYFWMNGDRIDSLLENDRQQTHMLDVMQDHFSRASSIIDELFQDRFFTREPQDTYHYLPFSLPHRRPHFFFPKS***GGGG*****SAWSHPQFEKGGGSGGGSGGSAWSHPQFEK*****GGGG***RIVRSLMPFSPYEPLNFHAMFQPFLEMIHEAQQAMDIHFHSPAFQHPPTEFIREGDDDRTVCREIRHNSTGCLRMKDQCDKCREILSVDCSTNNPSQAKLRRELDESLQVAERLTRKYNELLKSYQWKMLNTSSLLEQLNEQFNWVSRLANLTQGEDQYYLRVTTVASHTSDSDVPSGVTEVVVKLFDSDPITVTVPVEVSRKNPKFMETVAEKALQEYRKKHREE

3. *r*CLU-βN-ST

MMKTLLLFVGLLLTWESGQVLGDQTVSDNELQEMSNQGSKYVNKEIQNAVNGVKQIKTL
IEKTNEERKTLLSNLEEAKKKKEDALNETRESETKLKELPGVCNETMMALWEECKPCLKQ
TCMKFYARVCRSGSGLVGRQLEEFLNQSSPFYFWMNGDRIDSLLENDRQQTHMLDVMQD
HFSRASSIIDELFQDRFFTREPQDTYHYLPFSLPHRRPHFFFPKSRIVRS**_GGGG_SAWSHPQFE**
KGGGSGGGSGGSAWSHPQFEK_GGGG_LMPFSPYEPLNFHAMFQPFLEMIHEAQQAMDIH
FHSPAFQHPPTEFIREGDDDRTVCREIRHNSTGCLRMKDQCDKCREILSVDCSTNNPSQAKL
RRELDESLQVAERLTRKYNELLKSYQWKMLNTSSLLEQLNEQFNWVSRLANLTQGEDQY
YLRVTTVASHTSDSDVPSGVTEVVVKLFDSDPITVTVPVEVSRKNPKFMETVAEKALQEYR
KKHREE

4. *r*CLU-βC-ST

MMKTLLLFVGLLLTWESGQVLGDQTVSDNELQEMSNQGSKYVNKEIQNAVNGVKQIKTL
IEKTNEERKTLLSNLEEAKKKKEDALNETRESETKLKELPGVCNETMMALWEECKPCLKQ
TCMKFYARVCRSGSGLVGRQLEEFLNQSSPFYFWMNGDRIDSLLENDRQQTHMLDVMQD
HFSRASSIIDELFQDRFFTREPQDTYHYLPFSLPHRRPHFFFPKSRIVRSLMPFSPYEPLNFHA
MFQPFLEMIHEAQQAMDIHFHSPAFQHPPTEFIREGDDDRTVCREIRHNSTGCLRMKDQCD
KCREILSVDCSTNNPSQAKLRRELDESLQVAERLTRKYNELLKSYQWKMLNTSSLLEQLNE
QFNWVSRLANLTQGEDQYYLRVTTVASHTSDSDVPSGVTEVVVKLFDSDPITVTVPVEVS
RKNPKFMETVAEKALQEYRKKHREE**_GGGG_SAWSHPQFEKGGGSGGGSGGSAWSHPQF**
EK

Key for construct 5 (C-tag):

MMKTLLLFVGLLLTWESGQVLG: signal peptide sequence

EPEA: C-tag

5. *r*CLU-CT:

MMKTLLLFVGLLLTWESGQVLGDQTVSDNELQEMSNQGSKYVNKEIQNAVNGVKQIKTL
IEKTNEERKTLLSNLEEAKKKKEDALNETRESETKLKELPGVCNETMMALWEECKPCLKQ
TCMKFYARVCRSGSGLVGRQLEEFLNQSSPFYFWMNGDRIDSLLENDRQQTHMLDVMQD
HFSRASSIIDELFQDRFFTREPQDTYHYLPFSLPHRRPHFFFPKSRIVRSLMPFSPYEPLNFHA
MFQPFLEMIHEAQQAMDIHFHSPAFQHPPTEFIREGDDDRTVCREIRHNSTGCLRMKDQCD
KCREILSVDCSTNNPSQAKLRRELDESLQVAERLTRKYNELLKSYQWKMLNTSSLLEQLNE
QFNWVSRLANLTQGEDQYYLRVTTVASHTSDSDVPSGVTEVVVKLFDSDPITVTVPVEVS
RKNPKFMETVAEKALQEYRKKHREE**EPEA**

Supplementary Materials S2:

Immuno dot blot detection of *r*CLU-CT secreted by MEXi 293E cells

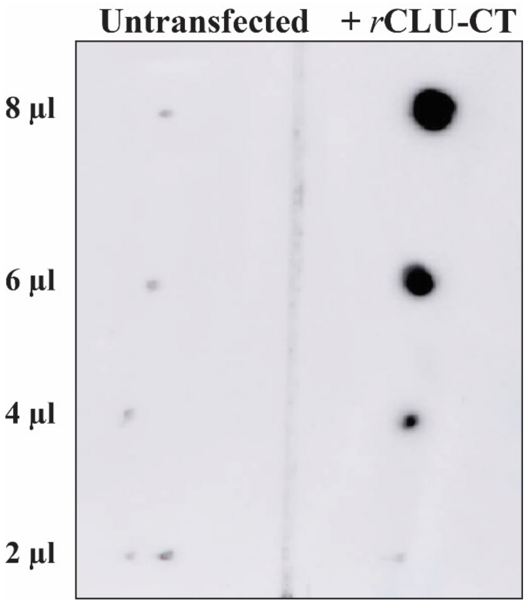

Figure S2 Immuno dot blot detection of *r*CLU-CT secreted by MEXi 293E cells. Culture supernatant (CS) from MEXi 293E cells overexpressing *r*CLU-CT was harvested on day 5 post transfection. Different volumes of CS (indicated at left side of image) taken from untransfected cells (grown for 6 days, left) or transfected cells expressing *r*CLU-CT (right) were spotted onto a nitrocellulose membrane. Specific immunodetection of *r*CLU-CT was performed as described in Methods. The image shown is representative of 2 independent experiments.

Supplementary Materials S3:

Complete SDS PAGE images corresponding to manuscript Figures 3B, 4A and 7A

A

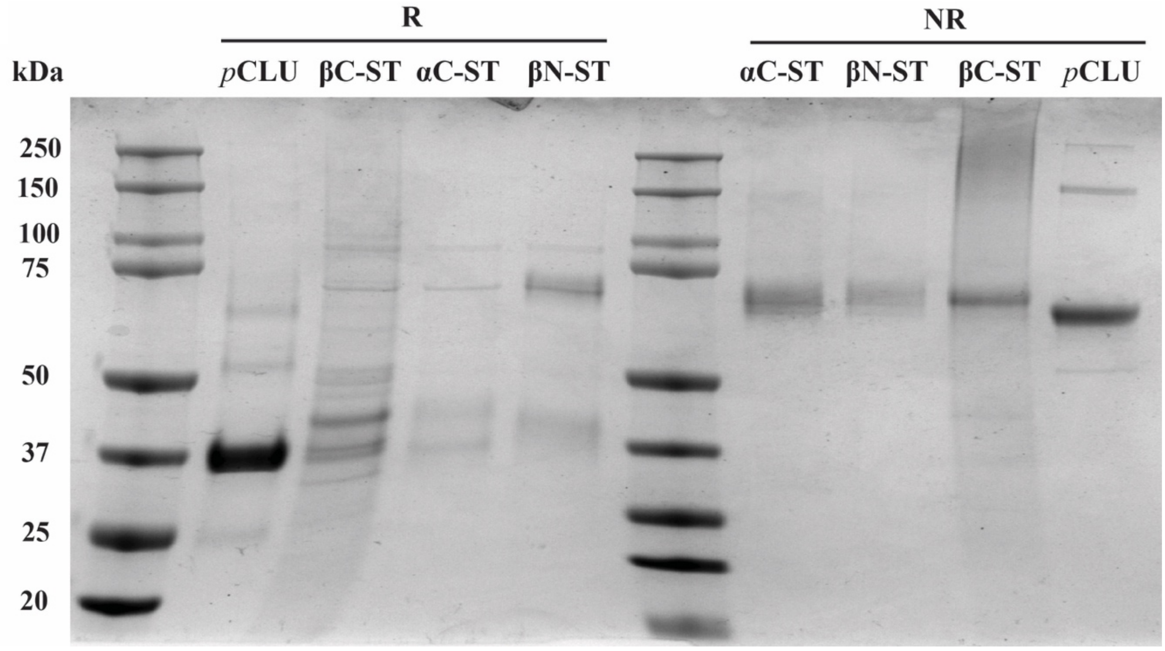

Figure S3A. Complete image of SDS PAGE of purified *r*CLU-βN-ST, *r*CLU-αC-ST, *r*CLU-βC-ST and *p*CLU. The molecular mass of protein standards (left lane) are shown in kDa. Reduced (R) and Non-reduced (NR) lanes are indicated above the image. Both panels shown in main text Figure 3B have been taken from this image.

B

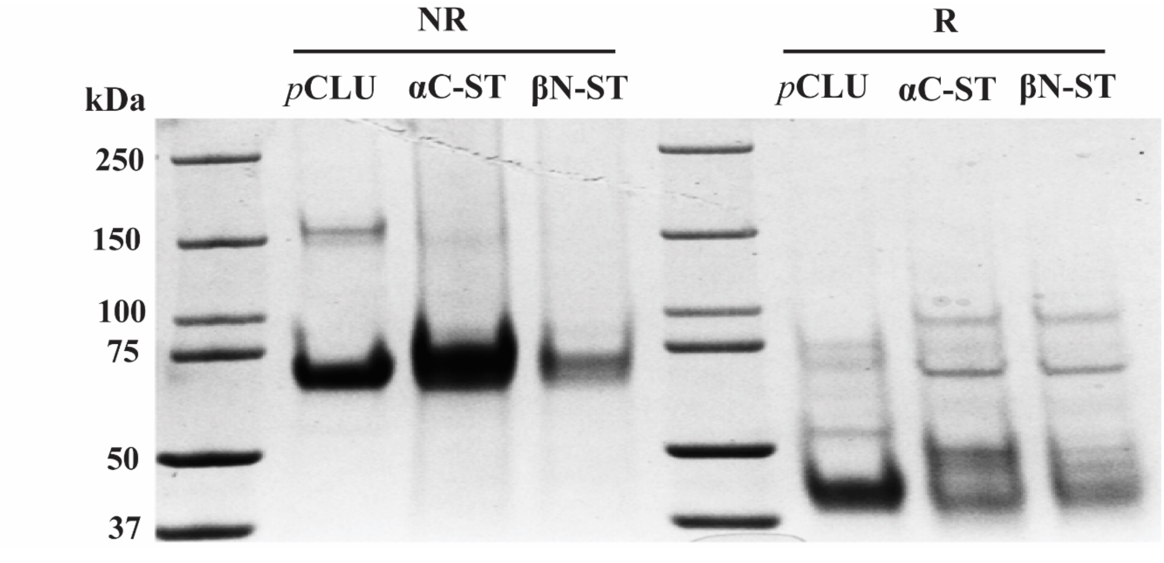

Figure S3B. Complete image of SDS PAGE analysis of purified *p*CLU, *r*CLU-αC-ST and *r*CLU-βN-ST under non-reducing (NR) and reducing (R) conditions. The molecular mass of protein standards (left lane) are shown in kDa. The left and middle panels in main text Figure 4A are taken from this image.

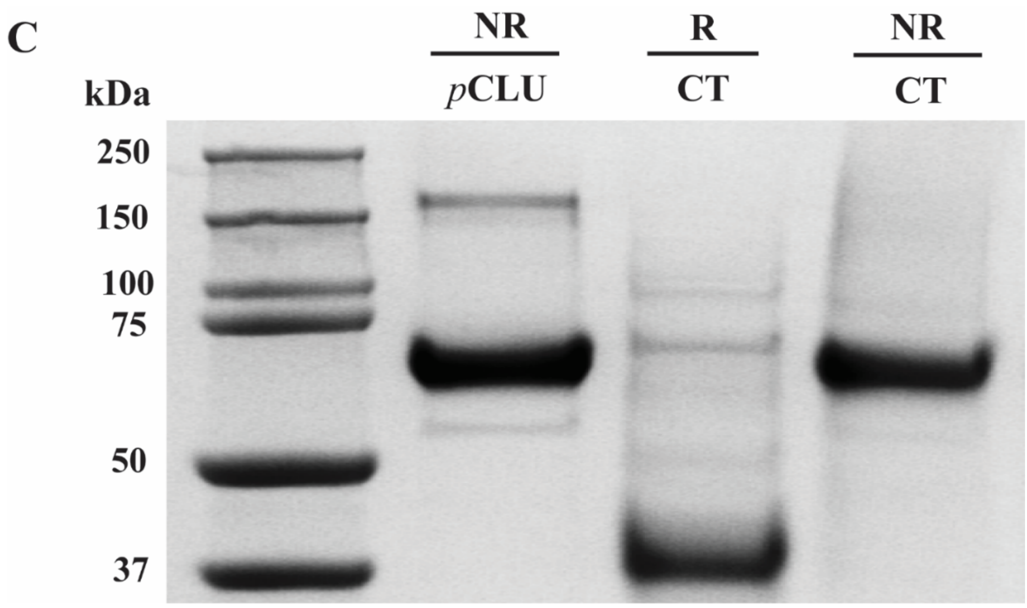

Figure S3C. Complete image of SDS PAGE analysis of purified *p*CLU, *r*CLU-CT under non-reducing (NR) and reducing conditions (R). The molecular mass of protein standards (left lane) are shown in kDa. The right panel in main text Figure 4A is taken from this image.

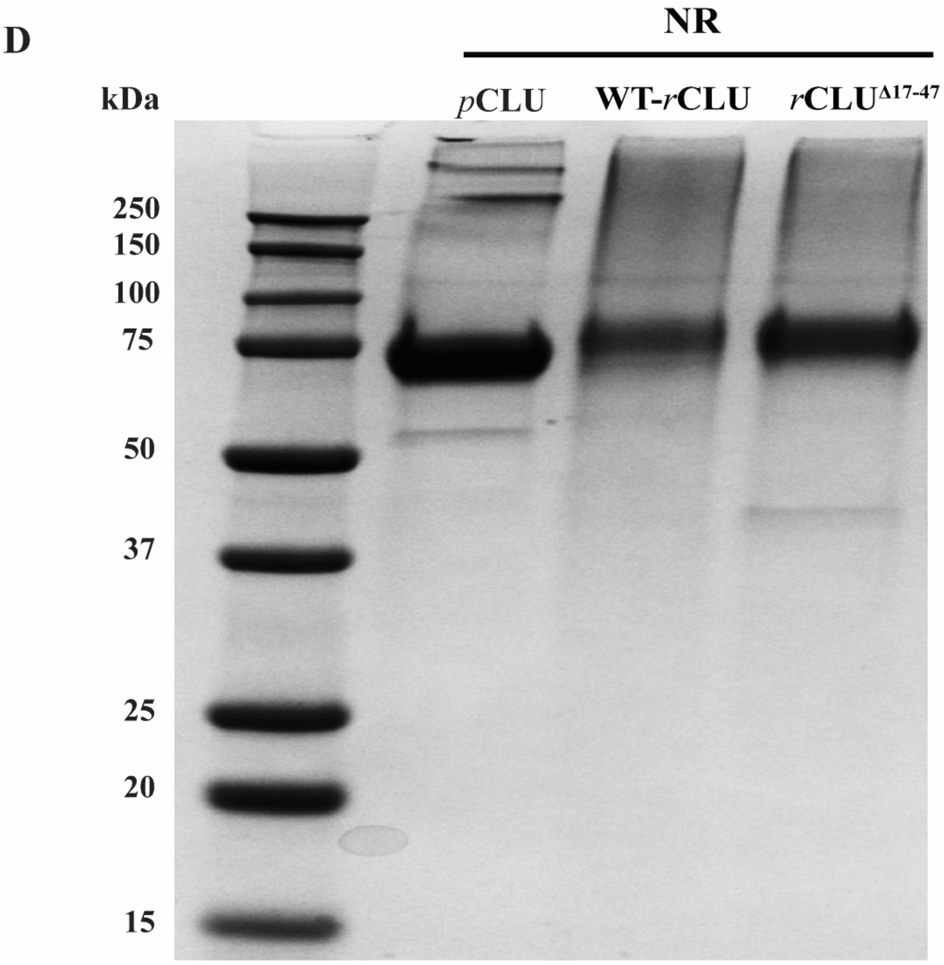

Figure S3D. Complete image of SDS-PAGE analyses of wild type and mutant *r*CLU under non-reducing conditions (NR). *p*CLU, full length WT-*r*CLU-αC-ST (WT-*r*CLU) and a CLU deletion mutant *r*CLU$^{\Delta 17\text{-}47}$-αC-ST (in which residues 17-47 were deleted) were analysed by SDS PAGE. The molecular mass of protein standards (left lane) are shown in kDa. The left panel in main text Figure 7A is taken from this image.

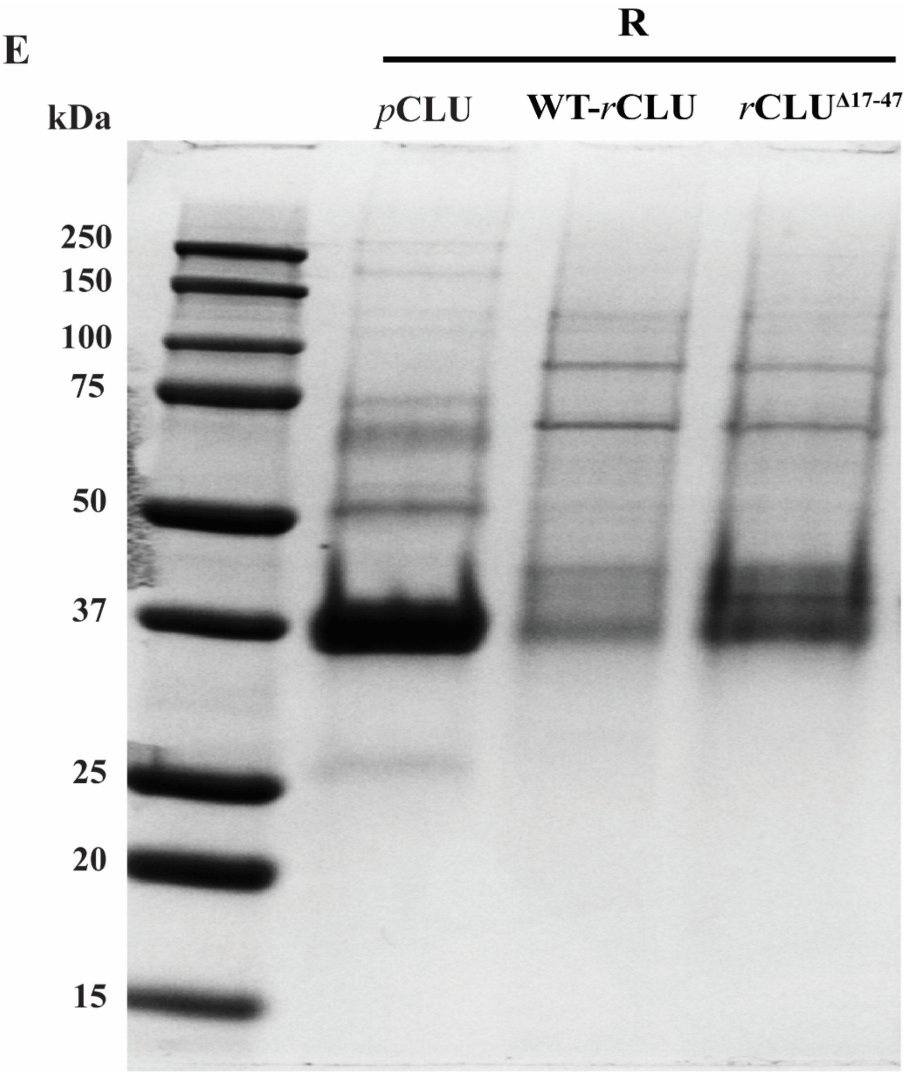

Figure S3E. Complete image of SDS-PAGE analyses of wild type and mutant *r*CLU under reducing conditions (R). *p*CLU, full length WT-*r*CLU-αC-ST (WT-*r*CLU) and a CLU deletion mutant *r*CLU$^{\Delta17-47}$-αC-ST (in which residues 17-47 were deleted) were analysed by SDS PAGE. The molecular mass of protein standards (left lane) are shown in kDa. The right panel in main text Figure 7A is taken from this image.
